# HMGB1: A Potential Target of Nervus Vagus Stimulation in Pediatric SARS-CoV-2-Induced ALI/ARDS

**DOI:** 10.3389/fped.2022.884539

**Published:** 2022-05-11

**Authors:** Lina Jankauskaite, Mantas Malinauskas, Goda-Camille Mickeviciute

**Affiliations:** ^1^Lithuanian University of Health Sciences, Medical Academy, Pediatric Department, Kaunas, Lithuania; ^2^Lithuanian University of Health Sciences, Medical Academy, Institute of Physiology and Pharmacology, Kaunas, Lithuania; ^3^Rehabilitation Center “Palangos Linas”, Palanga, Lithuania

**Keywords:** pediatric, SARS-CoV-2, COVID-19, ALI, HMGB1

## Abstract

From the start of pandemics, children were described as the ones who were less affected by SARS-Cov-2 or COVID-19, which was mild in most of the cases. However, with the growing vaccination rate of the adult population, children became more exposed to the virus and more cases of severe SARS-CoV-2-induced ARDS are being diagnosed with the disabling consequences or lethal outcomes associated with the cytokine storm. Thus, we do hypothesize that some of the children could benefit from nervus vagus stimulation during COVID-19 ARDS through the inhibition of HMGB1 release and interaction with the receptor, resulting in decreased neutrophil accumulation, oxidative stress, and coagulopathy as well as lung vascular permeability. Moreover, stimulation through alpha-7 nicotinic acetylcholine receptors could boost macrophage phagocytosis and increase the clearance of DAMPs and PAMPs. Further rise of FGF10 could contribute to lung stem cell proliferation and potential regeneration of the injured lung. However, this stimulation should be very specific, timely, and of proper duration, as it could lead to such adverse effects as increased viral spread and systemic infection, especially in small children or infants due to specific pediatric immunity state and anatomical features of the respiratory system.

## Introduction

Since the start of COVID-19 pandemics, children have been less addressed in the scientific investigations of SARS-CoV-2 infection as well as treatment implications. First, in comparison to adults' pediatric patients have been at lower risk for the severe course and lethal outcomes of COVID-19. Severe respiratory manifestations predominantly seen in adults were seldom documented in groups of pediatric patients who mostly tended to have an asymptomatic or mild form of infection (1–3). Mild COVID-19 in pediatric patients naturally leads to smaller hospitalization and death rates compared to sick adults. However, significant COVID-19 complications might occur in the pediatric age as well. With the increased mutation rate of SARS-CoV-2, the virus became more adaptable and targeted different age groups including children. Moreover, the pediatric population became more susceptible to SARS-CoV-2 infection due to increasing socialization, and more precautious enrollment in clinical vaccine trials resulting in later and slower vaccination rates (4). Overall, with the growing infectivity higher number of children are more likely to be severely ill, especially those with the risk factors (4). Still, the specific anti-SARS-CoV-2 treatment is lacking, and also, the pediatric population is less involved in the clinical trials for specific medication in case of severe COVID-19. Being a very specific part of the population with different immunity and distinct anatomy (e.g., respiratory tract) still under development over the whole childhood to the puberty, children require a specific glimpse into possible treatment applications during severe COVID-19 and SARS-CoV-2-induced acute lung injury (ALI).

Vagal nerve stimulation has been of interest in adult COVID-19 with a special focus on SARS-CoV-2-induced ALI. The basic concept behind is its anti-inflammatory activity with regard to cytokine storm reduction (5). Vagal nerve anti-inflammatory potential has been already shown in different chronic inflammatory processes, such as rheumatoid arthritis, systemic inflammation, acute postsurgical inflammatory response after lung lobectomy, or polymicrobial sepsis (6–9). In addition, in most the adult cases with a previous existing polymorbidity, COVID-19 is associated with decreased cardiac function. Thus, vagal stimulation can contribute to improvement via adjusting the sympathetic vagal imbalance (10). Children do have fewer cardiovascular and pulmonary comorbidities; thus, vagal nerve stimulation could be of interest emphasizing its immune-modulatory potential. First, it is demonstrated to limit dendritic cell recruitment into the lungs (11). Moreover, it can inhibit HMGB1 release, leading to a decrease of different pro-inflammatory cytokines. High-mobility group box protein 1 (HMGB1) has been studied as an initiating factor that participates in multiple inflammation-related diseases in adult and pediatric patients (12, 13). Research studies in the pediatric population show that many inflammatory diseases, including respiratory system-related diseases like pneumonia/bronchiolitis (14), influenza virus or respiratory syncytial virus (RSV)-induced infection, and lung injury (15, 16), correlate with increased levels of HMGB1. In the majority of cases, levels of HMGB1 are directly linked to the severity of the disease. Considering that SARS-CoV-2 might initiate inflammatory processes and induce ALI, HMGB1 could be used as a target for specific prevention and treatment option for pediatric COVID-19 and SARS-CoV-2-induced ALI. As nervus vagus activation can inhibit HMGB1 release, we hypothesize that vagal nerve stimulation could be of benefit in pediatric SARS-CoV-2 infection.

## Hypothesis Validation

### Acute Respiratory Diseases and Pathobiology of HMGB1

Different respiratory viruses, including SARS-CoV-2, target epithelial cells in the respiratory tract and through their cytopathic effect induces cellular death leading to a local release of different damage-associated molecular pattern proteins (DAMPs) together with pathogen-associated molecular pattern proteins (PAMPs) (17). High mobility group box 1 (HMGB1) is one of the most broadly studied DAMPs which is upstream of interleukin (IL) 6 release (13, 18). It plays an important role in different acute and chronic lung diseases as well as other inflammatory processes caused by various stimuli (19–21). HMGB1 plasma levels are shown to be associated with severity of the disease, survival, and mortality of bacterial pneumonia complicated by ARDS, meanwhile, treatment with HMGB1-specific antagonist revealed improved survival in preclinical animal models of acute inflammation (22–24). The significantly increased HMGB1 levels were detected in bronchoalveolar lavage fluid prior to the severe hyperoxia-induced lung injury (25). In a study of neonatal ARDS (NRDS), HMGB1 is considered a crucial indicator of disease severity and clinical outcomes (26, 27). Few studies proved that RSV, a leading cause of infant bronchiolitis, promotes HMGB1 release and respiratory epithelial cell necroptosis (28–30).

HMGB1 is a chromatin-linked, non-histonic protein, 99% identical to mammals with a cytokine activity on a cytosolic, nuclear and extracellular level (18). After damage and cellular death or via active secretion by stimulation of innate immune cells, excessive quantities of HMGB1 are released resulting in an inflammatory process together with enhanced cells, such as neutrophils or monocyte, recruitment, migration, and facilitated cell proliferation. Experimental studies demonstrated that HMGB1 plays a pivotal role in ALI through the recruitment of leucocytes into the lungs (25, 31). In addition, it induces neutrophil dysfunction in experimental sepsis models (32). Moreover, HMGB1 stimulates dendritic cell maturation and enhances antibody response (33, 34). The HMGB1 triggered inflammatory process is generated in two ways, i.e., binding HMGB1-specific receptors or forming complexes with DNA, RNA, or other DAMPs (35). Extracellular HMGB1 binds to Toll-like receptors (TLR), such as TLR3, TLR4, and receptors for the advanced glycation end product (RAGE). Thus, the P38 mitogen-activated protein kinase (MAPK), extracellular signal-regulated kinase 1/2 (ERK1/2), nuclear factor (NF)-kB, and other downstream pathways are activated which results in the secretion of tumor necrosis factor (TNF) α, IL-1β, IL6, transforming growth factor (TGF)-1β, platelet-derived growth factor (PDGF) and other molecules, leading to increased tissue damage and organ dysfunction (36, 37). HMGB1-RAGE/TLR4-axis has been attributed to a central role in influenza virus-induced infection models. Additionally, in experimental studies, HMGB1 antagonists demonstrated partial protection against influenza-induced lung injury as well as encephalopathy (38, 39), and prevention of necroptotic respiratory epithelial cell death (40). There are growing data regarding HMGB1 function in the regulation of autophagy and its diagnostic potential in ALI (41–43). HMGB1 is a late mediator causing cell apoptosis and autophagy via translocation of NF-kB inducing the production of various previously mentioned pro-inflammatory cytokines (44). Different cytokines further stimulate the release of HMGB1, thus, contributing to a positive feedback loop enhancing the inflammatory cascade (25, 45).

### HMGB1 Role in SARS-CoV-2-Induced Lung Inflammation

Considering previous findings, we hypothesize that HMGB1 can be a crucial player in SARS-CoV-2-induced lung injury of adults as well as in pediatric patients. Indeed, a study by Chen et al. revealed that COVID-19 patients had higher plasma levels of HMGB1 compared to healthy volunteers (46). In SARS-CoV-2-induced respiratory tract infection, HMGB1 initiates inflammation via two different pathways. First, it triggers TLR4 leading to IL6 release. IL6 is one of the essential pro-inflammatory mediators in COVID-19 infection. Increased plasma levels of IL6 are detected in approximately half of patients with COVID-19 and are associated with the disease severity (47, 48). A study by Sivakorn et al. detected a significant correlation between HMGB1 and IL6 levels and other prognostic biomarkers, such as D-dimer and C-reactive protein (CRP) on admission to intensive care unit (ICU) (49). Even though, most children present asymptomatic or with the mild COVID-19 showing normal concentrations of IL6 (50), severely ill patients do have increased values of IL6 (51). Moreover, IL6 is frequently elevated in MISC – a multisystemic inflammatory response syndrome that is associated with a previous asymptomatic SARS-CoV-2 infection (52). A study by Abrams et al. demonstrated that higher IL6 levels together with other factors such as CRP or ferritin were linked to increased MISC admission to pediatric ICU, and IL-6 concentrations were related to shock (53). Taking into account above mentioned experimental data and clinical studies from the adult population, we speculate that HMGB1 could be an important contributor to pediatric COVID-19. The data are supported by the fact that HMGB1 is associated with RSV-induced bronchiolitis, neonatal ARDS, pediatric asthma, and pneumonia (12).

The second mechanism of HMGB1 action is to promote the expression of angiotensin-converting-enzyme 2 (ACE2) receptor on respiratory epithelial cells (13). ACE2 is a crucial host receptor for the entry of SARS-CoV-2 and is widely expressed in the human body, including naso- and oropharyngeal mucosa, and lower respiratory tract, e.g., alveolar epithelial cells. HMGB1 induces ACE2 expression via RAGE (54) which is important in different respiratory conditions as well as ALI/ARDS (55). As shown in a study by Pinto et al., increased expression of ACE2 correlates with SARS-CoV-2 induced disease severity and outcome (56). Few studies identified lower levels of ACE2 expression in children compared to adults, thus, it could partially explain milder COVID-19 cases in the pediatric population (57–59). However, some of the data show, that ACE2 expression could be ethnicity dependent (60). Moreover, ACE2 expression in children positive for SARS-CoV-2 was higher compared to negative ones (59, 61). Nevertheless, the ACE2 expression level was not related to viral loads (59). Thus, the mechanism of HMGB1-ACE2 could be of less importance in pediatric COVID, still, it could be of interest in severe SARS-CoV-2 induced ALI or in specific risk populations.

### ALI, HMGB1, and Cholinergic System

**Figure 1:**
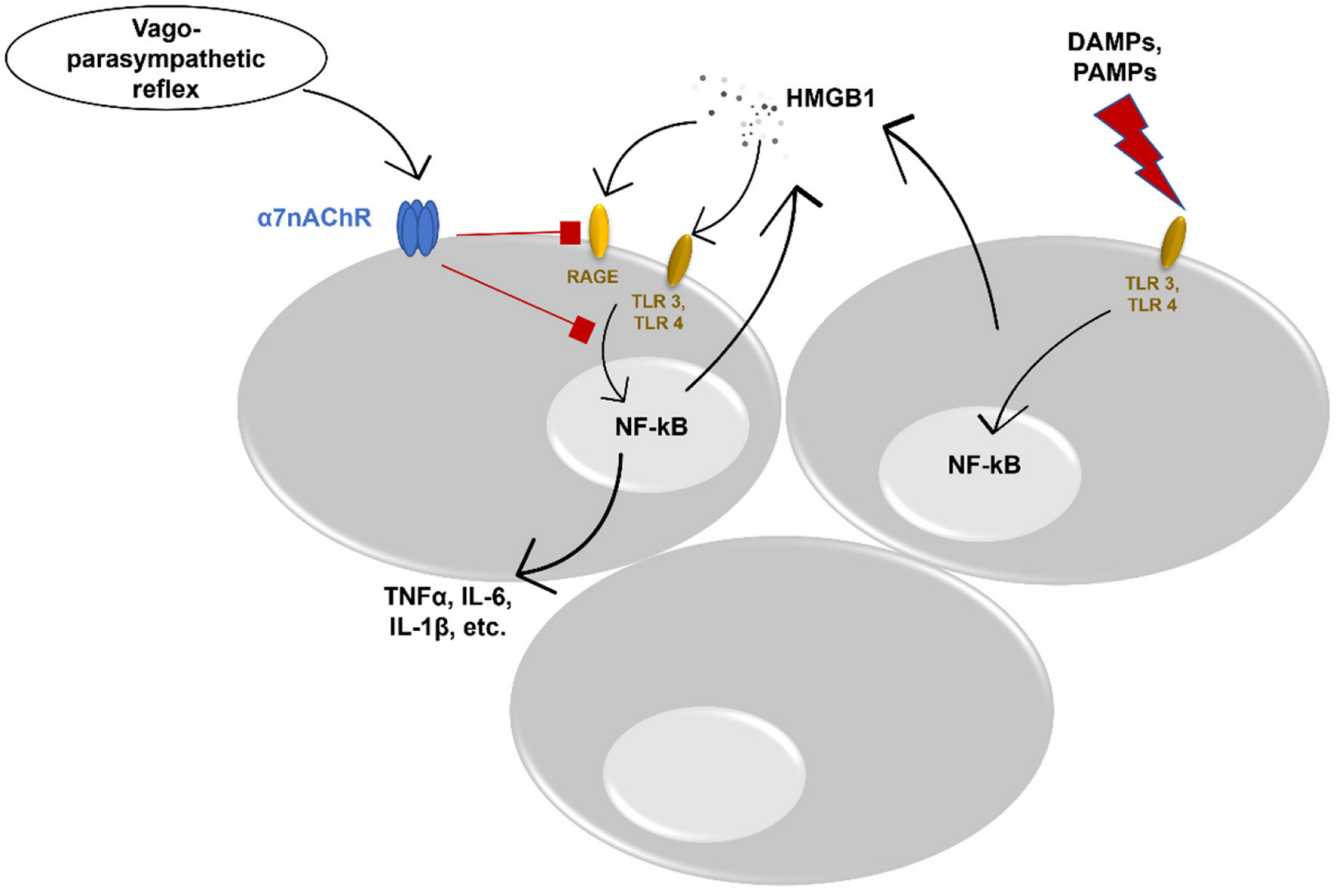
Potential anti-inflammatory effect of vagus nerve stimulation. DAMPs, danger associated molecular patterns; PAMPs, pathogen associated molecular patterns; TLR, toll-like receptor; NF-kB, nuclear factor kappa-light-chain-enhancer of activated B cells; HMGB1, High-mobility group box protein 1; RAGE, receptor for advanced glycation end product; TNFα, tumor necrosis factor alpha; IL, interleukin; α7nAChR, alpha 7 nicotinic acetylcholine receptor.

A growing body of evidence shows the interplay between different immune cells, pro-inflammatory cytokines, and chemokines, and neural cells in the respiratory tract (5). Vagal afferents in the lung can identify different tissue-damaging factors (62) and contribute to the reduction of inflammation via e.g., the cholinergic anti-inflammatory pathway (63, 64). As the major sensory channel in the airway-brain axis, the vagus nerve regulates respiration, and respiratory defense and provides “feedback” to the brain (65). This process can be disturbed via ALI/ARDS. More data demonstrate an important role of the cholinergic system in ALI via inhibition or reduction of HMGB1. A recent study by Sitapara et al. revealed a potential therapeutic effect of alpha 7 nicotinic acetylcholine receptor (α7nAChR) agonist which attenuated hyperoxia-induced acute lung injury (HALI) by a significant decrease of HMGB1 levels in the airways and serum of mice (66). Nevertheless, the same study showed improved macrophage phagocytosis. Induction of α7nAChR demonstrated to prevent activation of NF-kB pathway leading to inhibition of HMGB1 secretion in *in vitro* cultured human macrophages (67). In addition, in experimental mouse models, vagal nerve stimulation reduced serum HMGB1 levels resulting in increased survival (68). The cholinergic anti-inflammatory pathway affects HMGB1 release and inhibits HMGB1 receptor-mediated activities. Acetylcholine and α7nAChR agonist constrained RAGE-induced endocytosis of HMGB1 complexes leading to downregulation of proinflammatory cytokines and pyroptosis (69). Moreover, α7nAChR signaling inhibits inflammasome activation, thus, reducing the release of HMGB1 together with IL-1α, IL-1β, and IL-18 (70) (Figure 1). In addition, vagal-α7nAChR stimulation promotes lung stem cell proliferation and differentiation in FGF-10 dependent manner (71). FGF-10 is important in mesenchymal stromal cell mobilization and lung inflammatory cytokines' reduction. Altogether, vagal-α7nAChR stimulation in pediatric patients could dampen SARS-COV-2-induced lung inflammation and promote lung tissue recovery via inhibition of HMGB1 and FGF-10 induction.

## Application of Nervus Vagus Stimulation in Children

To date, different studies and protocols discuss and propose the best techniques and modes of vagal nerve stimulation (72). The duration and mode of nervus vagus stimulation seem to be pathology and age-dependent with a focus on a preferable effect. In addition, there are some other important confounding factors, such as gender, time of the day, and concomitant diseases of a patient. The majority of the studies apply transcutaneous, or percutaneous auricular nervus vagus stimulation associated with minimal side effects, such as local irritation or pain (72). A short-term auricular vagal stimulation has been urged for COVID-19 ALI patients as well (5, 73). Considering the pediatric population, transcutaneous auricular stimulation would be most suitable as this application is non-invasive with minimum transient side effects. Moreover, children do have less confounders, as they do have fewer comorbidities, thus, they are using fewer medications which could interfere with the beneficial effect of n. vagus stimulation or influence mode and duration of its application. Nevertheless, a lot of severely ill pediatric COVID-19 patients have different comorbidities (74, 75) and could be prescribed various medications, such as anti-inflammatory agents (non-steroid or glucocorticoids), etc. Thus, vagal stimulation modifications emphasizing different duration and frequencies with regard to the specific side effects and COVID-19 treatment applications might be taken into account. However, nervus vagus stimulation in neonates, premature babies, and infants, which correspond to another group of pediatric patients prone to severe COVID-19, could be challenging due to their anatomy, i.e., smaller ears. Overall, additional even non-invasive procedures applied in a healthcare environment can be stressful and children, especially smaller ones, can be less compliant compared to adults (72). Still, vagal stimulation has been successfully used in different pediatric conditions, such as nephrotic syndrome or epilepsy (76–79), showing its potential and opportunities in other pediatric diseases, including SARS-CoV-2-induced ALI. Another consideration should be with regard to voltage and frequency of nervus vagus stimulation. The higher voltage could be associated with pronounced bronchospasm (80), thus low voltages should be applied, especially in systemic nervus vagus stimulation, as specific fiber stimulation in case of lung pathology or specific stimulation of α7nAChR might be challenging. In addition, children do have different normal physiological heart rates (HR) and it is age-dependent. HR is shown to predict vagal nerve stimulation response (81), hence, it would be important to consider when applying vagal stimulation in children of different ages. The frequency and duration must be sufficient to decrease SARS-CoV-2-induced inflammation and be tolerable for a child. Higher voltages and longer duration can be associated with more side effects, including local pain. Currently, there is no clear duration and frequency for nervus vagus stimulation in acute pediatric conditions. Nevertheless, 2-h stimulation with 2 h off for few days (79) or once a day for 5 min (76) has been described, still, it could not be translated to COVID-19. Considering the pathophysiology and immunology of COVID-19, vagal stimulation might be started in a later phase of SARS-CoV-2-induced infection and for few days. However, there is increasing data that neuromodulation could benefit symptoms of long-covid (82) which is more frequent in asymptomatic children or children with mild disease (83), thus repeated stimulation could be applied to improve post-covid signs and symptoms.

## Possible Risks of Vagal Nerve Stimulation in Pediatric COVID-19

In a variety of model systems, the vagus nerve acts as an anti-inflammatory. The majority of these counter-inflammatory actions have been attributed to macrophage nicotinic receptor activation (84–87). Little is known about the role of the vagal nerve in modulating the activity of other cells, such as CD4+ T cells and CD8+ cells, involved in an inflammatory response. SARS-CoV-2 infection may not be unique in this aspect as most the acute viral infection pathogens induce both CD4+ and CD8+ cells activation and proliferation (88). However, activation of T-cells might not be optimal in some patients with COVID-19, due to delayed or defective type I interferon (IFN) responses (89), potentially distorting T cell responses. Type I interferon (IFN-alpha and IFN-beta) is secreted by virus-infected cells (90), and this explains why viral load could have a major impact on the magnitude and quality of the T cell response (91). Besides, even though infants have a good cellular arsenal fighting against viral infections, they can still be underdeveloped (92). As an example, newborns do have diminished production of interferons which reaches adult levels only in children who are 1–2 years old. Furthermore, the production of other pro-inflammatory cytokines is decreased, and levels are rising with age, however, still lower than in adults (93). Additionally, neonates do present with an impaired macrophage response to IFN-gamma and neonatal monocytes and macrophages do have impaired response to multiple TLR ligands, which are crucial in anti-viral and initial pro-inflammatory response (94). Moreover, infants do have an attenuated capacity to generate memory CD4+ T cell response (94). Thus, they are physiologically and immunologically more prone to viral infections with a possibility of severe complications (e.g., pneumonia, ALI/ARDS). Reduced T cell response dependent on IFN also has been ascribed to the activation of the vagus nerve (95, 96). T cells play critical roles in pulmonary host defense against viral pathogens therefore stimulation of the vagal nerve would affect the initial response to the pathogen, which allows the virus to spread further bypassing the primary barrier. CD4+ (helper) cells help B cells to produce antibodies promoting the long-standing protective immunity (97), and, thus, the stimulation of the vagus nerve during the incubation period and in the early stages of the symptoms appearance of coronavirus disease is not recommended. One prominent feature of SARS-Co-V-2 infection is lymphopenia with the following decrease in the count of CD4+ T cells, CD8+ T cells, B cells, and natural killers (98, 99). Many respiratory viruses, such as influenza or human rhinovirus, cause transient lymphopenia, which lasts only 2–4 days after symptoms start (100), while COVID-19 associated lymphopenia may be more severe or persistent (101). The peripheral lymphopenia observed in patients with COVID-19 may reflect the recruitment of lymphocytes to the respiratory tract, although autopsy studies of patients' lungs were not identified excessive levels of lymphocytes in bronchoalveolar lavage fluid (102). The mechanism of lymphopenia in COVID-19 remains incompletely understood, therefore, there is a presumption that the reduction in the number of T cells may be related to the anti-inflammatory role of the vagus nerve.

Dendritic cells (DC) are potent antigen-presenting cells that respond to sites of injury and are crucial for the priming phase of the immune response (103). Vagal nerve stimulation produces an anti-inflammatory effect by inhibiting pulmonary DC recruitment to the lung and preventing ALI in animal models (11, 104). Neonatal DCs are less polyfunctional than those of adults, in response to TLR activation, therefore neonates and infants are much more susceptible to a wide variety of infections (105). As compared to adults, this higher susceptibility is speculated to reflect impairments in both innate and adaptive immunity (106). On activation, pulmonary DC produces a range of inflammatory mediators (107), including the cytokines interleukin (IL)-6 and transforming growth factor (TGF)-beta (108, 109). TGF is a key mediator of pulmonary edema in ALI as it is triggered locally by the αvβ6 integrin (110). DC cells are also known as the IFN factory of the immune system, equipped to detect viruses and able to produce IFN levels far exceeding those produced by infected cells or other immune cells (111). Understanding the density and activity of DCs in COVID-19 is critical due to their potential to significantly amplify the IFN activation, which also represents a risk for immunopathology (112). Moreover, there are findings suggesting reduced IFN signatures in patients with severe COVID-19 (113), in contrast to the early and strong IFN responses found in antiviral responses of mild SARS-CoV-2 infection (114). Based on these facts, it is conceivable that stimulation of the vagus nerve may weaken the antiviral barrier in adults, and may have an even greater effect in infancy, as most dendritic cells are immature (115).

The afferent and efferent vagus nerve, α7nAChR-expressing inflammatory cells, and the central vagal nucleus in the brain form an inflammatory reflex that could finely coordinate inflammation and immunity (116). Activation of this pathway is dependent on the level of inflammation in the local lung tissue (86), therefore the cholinergic anti-inflammatory pathway alleviates acute inflammation in turn preventing ALI (117). Agonists to α7nAChR reduce the release of extracellular HMGB1 and expression of the two main HMGB1 receptors RAGE and TLR4 and thus inhibit HMGB1-driven inflammation (118). It seems this cholinergic anti-inflammatory reflex is highly responsible for HMGB1 expression, and it is locally regulated, therefore the effect of external stimulation of the vagal nerve can be debatable. In addition, the study suggests, that vagus nerve stimulation significantly reduces TNF (tumor necrosis factor) levels in the spleen (94%) and liver (40%) but not in the lung (20%) (119), which explains external vagal stimulation influence is more systemic rather than local on leukocytes and it secreted cytokines level. Hence, it is possible, that the external vagus nerve stimulation may not be less effective in children, and the therapeutic effect might be achieved through the changes in systemic immunity.

Taken all together, nervus vagus stimulation could be an optimal therapeutic option in pediatric patients, however, a specific population of children should be selected, e.g., older children and adolescents could benefit more compared to neonates or infants due to their immune systems which is more mature and closer to adults. Moreover, a specific period of COVID-19 must be considered as well. Children could be less capable of effectively responding to vagal nerve stimulation during the early phase of SARS-CoV-2-induced infection and it can lead to a boosted viral spread with a systemic disease due to dampened antiviral response which is already immature.

## Data Availability Statement

The original contributions presented in the study are included in the article/supplementary material, further inquiries can be directed to the corresponding author.

## Author Contributions

LJ: hypothesis, editing, and supervision. LJ, MM, and G-CM: analysis, writing original draft, and review. All authors contributed to the article and approved the submitted version.

## Conflict of Interest

The authors declare that the research was conducted in the absence of any commercial or financial relationships that could be construed as a potential conflict of interest.

## Publisher's Note

All claims expressed in this article are solely those of the authors and do not necessarily represent those of their affiliated organizations, or those of the publisher, the editors and the reviewers. Any product that may be evaluated in this article, or claim that may be made by its manufacturer, is not guaranteed or endorsed by the publisher.
